# Utility of synthetic hybrid-scale fiber matrix in dermatologic wounds

**DOI:** 10.1016/j.jdcr.2023.08.020

**Published:** 2023-08-29

**Authors:** Martin Zaiac, Matthew Kauffman, Taraneh Matin, Jordan Rosen, Emily Sallade

**Affiliations:** a Chairman, Department of Dermatology, Herbert Wertheim College of Medicine, Florida International University, Miami, Florida; b Director, Greater Miami Skin and Laser Center, Mount Sinai Medical Center, Miami Beach, Florida; c Greater Miami Skin and Laser Center, Mount Sinai Medical Center, Miami Beach, Florida; d Mohs and Cosmetic Dermatology Fellow, Hollywood Dermatology and Cosmetic Surgery Specialists, Hollywood, Florida; e PGY-4 Resident, Dr Phillip Frost Department of Dermatology and Cutaneous Surgery, University of Miami Miller School of Medicine, Miami, Florida; f Clinical Research Coordinator, Acera Surgical Inc, St. Louis, Missouri

**Keywords:** dermatologic wounds, post-Mohs wounds, scalp wounds, synthetic hybrid-scale fiber matrix, wound healing

## Introduction

Nonmelanoma skin cancers, including basal cell carcinomas and squamous cell carcinomas, are commonly diagnosed and often managed via Mohs micrographic surgery.^1^ This procedure removes a thin margin of tissue around and deep to the carcinoma until the margins are negative for tumor involvement, which may result in a large defect.^2^^,^^3^

Patient age, comorbidities, and resection size and location can complicate post-Mohs wound management.^2^^,^^3^ Large post-Mohs scalp defects are often managed with graft, flap, or linear reconstruction, and occasionally secondary intent healing.^2^ Scalp defects are particularly challenging, given the need for a well-granulated wound bed to ensure graft survival and high rates of flap necrosis among the elderly population.^2^ Linear repairs are commonly performed as well but are typically associated with a fewer number of stages.^4^

A synthetic hybrid-scale fiber matrix (SHSFM) could present a novel treatment option in managing post-Mohs scalp wounds. The SHSFM (Restrata, Acera Surgical, Inc) is a synthetic wound healing matrix that resembles human extracellular matrix in size and structure.^5^ This engineered design encourages cellular ingrowth through topographical cues, and then resorbs at a rate matching cellular proliferation with minimal inflammatory response.^5^

## Case report

Two clinical cases assessing the SHSFM for treatment of post-Mohs scalp wounds were conducted via a retrospective review of patient charts at a single site. Both patients consented to treatment and wound photographs were deidentified.

Each carcinoma was excised until negative margins were achieved. The SHSFM was then fenestrated and trimmed to wound size, applied in full contact with the wound bed, and secured with sutures or adhesive strips. A nonadherent silicone dressing was applied over the matrix and a bolster was sutured down over the dressing. Hydrogel was applied if clinically indicated. Healing was monitored at follow up visits, and the SHSFM was reapplied as clinically indicated until complete wound closure.

### Patient 1

**Fig 1:**
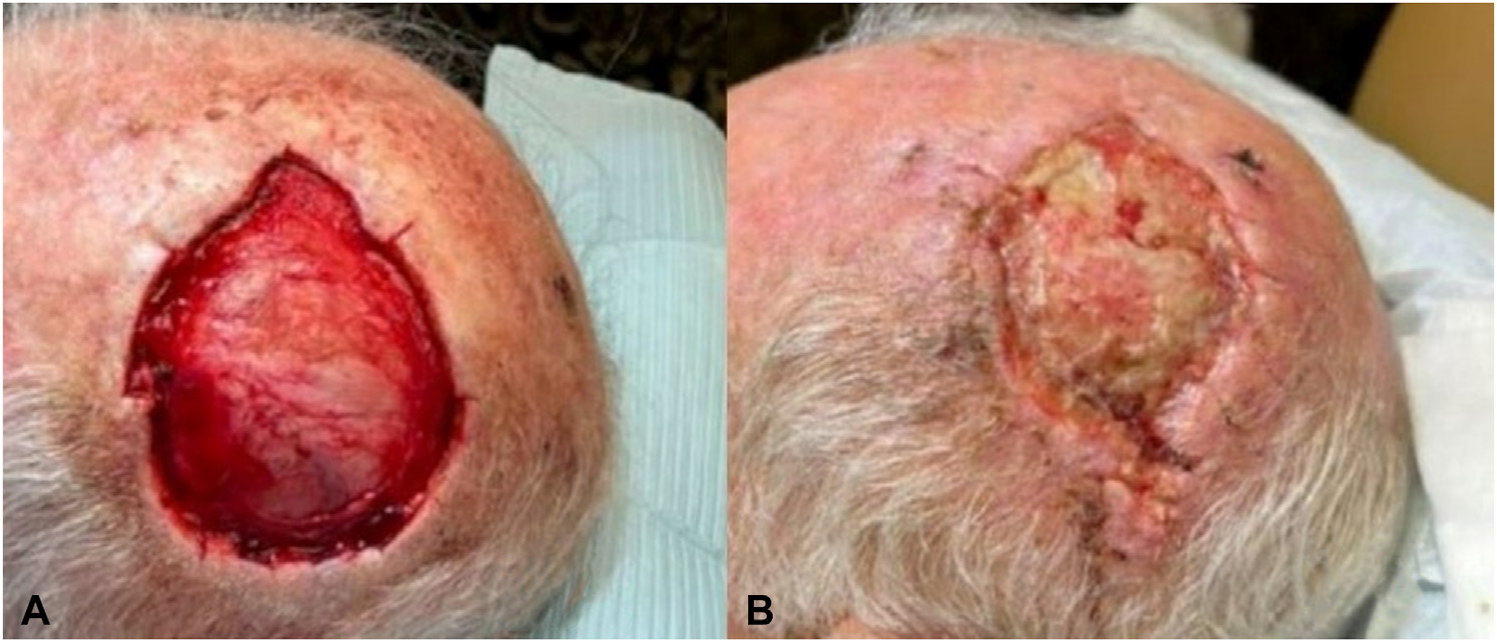
**A,** The initial post-Mohs defect. **B,** The post-Mohs wound at day 19. The matrix can be observed resorbing into the wound bed.

A 79-year-old male presented with a squamous cell carcinoma on the scalp measuring 3.6 cm × 2.8 cm at biopsy and was referred for Mohs micrographic surgery. Negative margins were obtained after 5 stages (Fig 1, *A*). The post-Mohs defect measured 9.2 cm × 7.6 cm with exposed bone and periosteum. Postoperatively, the patient returned for SHSFM application. The matrix was fenestrated, trimmed, applied to the wound bed, and sutured in place. Hydrogel was applied to maintain moisture, and a nonadherent primary dressing was applied over the matrix. A dental roll bolster was prepared and sutured down over the dressing.

Significant regranulation was observed as the matrix continued to resorb at the day 19 visit. (Fig 1, *B*). One week later, the wound measured 4.8 × 3.0 cm and the SHSFM had completely resorbed. A second piece of the matrix was fenestrated and applied. The SHSFM was secured with adhesive strips and applied in conjunction with hydrogel and a bolster dressing. At days 39 and 46, the SHSFM continued to resorb, and reepithelialization was noted around wound edges. At day 68, the wound had fully reepithelialized.

### Patient 2

**Fig 2:**
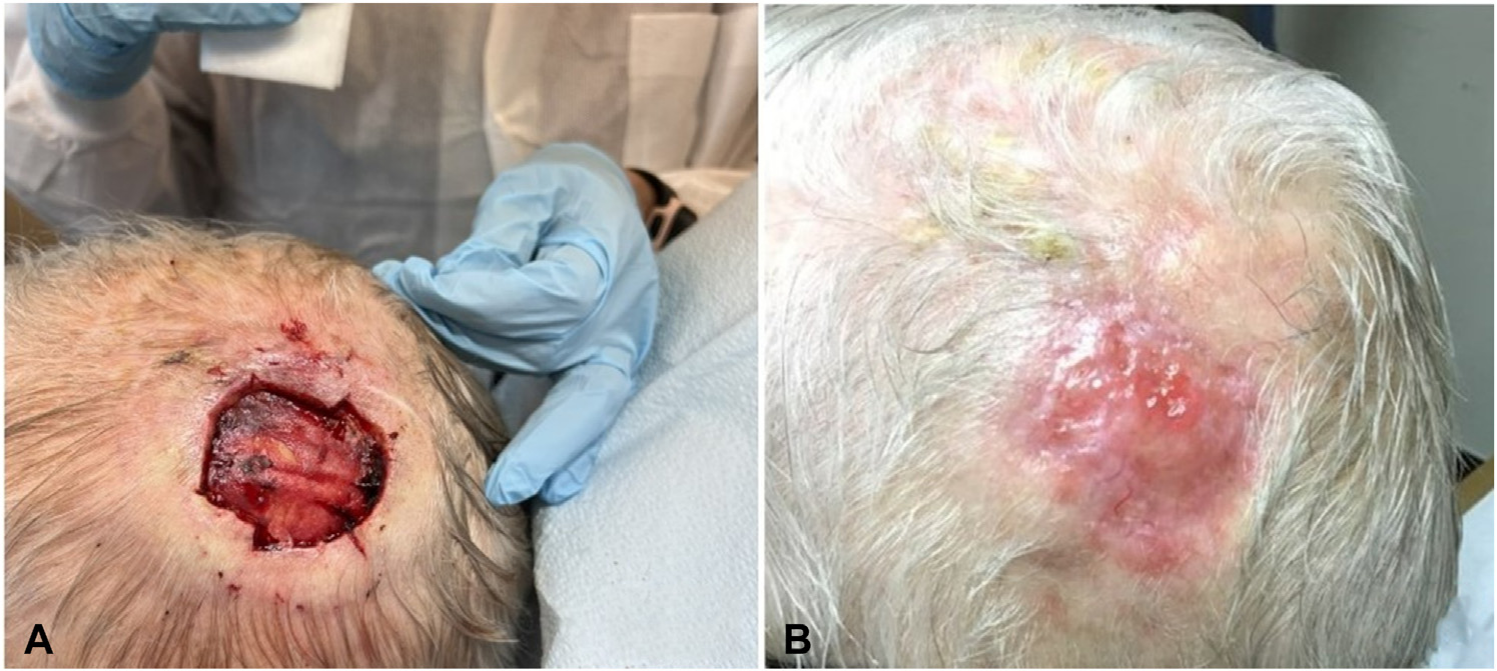
**A,** The postoperative Mohs defect. **B,** The post-Mohs surgical defect fully reepithelialized at day 81.

An 89-year-old male presented with a basal cell carcinoma on the scalp measuring 2.6 cm × 2.2 cm at biopsy. The patient underwent Mohs micrographic surgery, and negative margins were obtained after 8 stages. The post-Mohs defect measured 5.8 cm × 5.4 cm with exposed bone and periosteum (Fig 2, *A*). The SHSFM was not available at the facility at the time of the procedure and was applied after 4 days postoperatively in conjunction with hydrogel and a bolster dressing.

The patient returned 2 weeks after application for dressing changes and wound monitoring. Three weeks after initial application, a second piece was applied, as the initial application had resorbed. The matrix was meshed, secured to the wound bed with adhesive strips, and covered in a non-adherent primary dressing. The patient was seen at week 4 for wound assessment and dressing changes.

At week 6, the SHSFM was resorbing and was left in place. At week 7 the wound bed was fully granulated, and reepithelialization was observed at wound edges. By week 12, the wound was fully closed (Fig 2, *B*).

## Discussion

Large, deep post-Mohs defects are often closed primarily with flaps or skin grafts, but this may not be the best option dependent on the patient or wound. In the present 2 case studies, a SHSFM was utilized to stimulate granulation tissue formation and subsequently reepithelialize large post-Mohs scalp wounds. Both patients were satisfied with the cosmetic result and minimal at home wound care required.

Grafts require additional surgery and elderly patients are at risk for graft failure or necrosis.^2^ Secondary intent healing is an option for these patients but results in prolonged heal times. Previous studies assessing secondary intent heal time in post-Mohs scalp wounds with exposed bone and periosteum have demonstrated an average time to reepithelialization of 186 days.^6^

Prior studies have demonstrated that utilizing advanced wound therapies in post-Mohs wounds may improve healing times. A 1-stage reconstruction study of a dermal regeneration template demonstrated an average heal time of 42.5 ± 6.4 days.^7^

The results observed in the present case reports demonstrate vastly improved heal times when compared to healing via secondary intent alone. The average time to heal in the present 2 cases was 74.4 ± 6.5 days. The dermal regeneration template study included wounds with mean areas of 12.51 cm^2^ and 28.7 cm^2^.^7^ Wound sizes in the present case reports measured 69.9 cm^2^ and 31.3 cm^2^, which healed in 68 days and 81 days respectively.

This study has a few limitations, such as the retrospective design, limited number of patients, and lack of a comparison or control group. Additional work should be considered to further evaluate outcomes observed here.

The success of the SHSFM observed here is likely due to the design of the matrix, which encourages cellular infiltration and neovascularization by replicating human extracellular matrix.^5^ Prior studies utilizing the SHSFM in post-Mohs defects have demonstrated minimal scarring and excellent aesthetic results.^8^ The SHSFM could be a viable alternative to flaps and grafts in elderly patients and may limit the risk of poor cosmetic outcomes and prolonged heal times seen with secondary intent alone.

## Conflicts of interest

Author Sallade declares employment and stock options from Acera Surgical, Inc. Dr Zaiac, Authors Kauffman, Matin, and Dr Rosen have no conflicts of interest to declare.
